# Incidence of growth disturbance after distal tibia physeal fracture in children

**DOI:** 10.1186/s13018-022-03427-4

**Published:** 2022-12-08

**Authors:** Hui Chen, Zhao Chen, Peisheng Chen, Zibing Zheng, Jinrun Lin

**Affiliations:** 1 Department of Pediatric Orthopedics, Fujian Maternity and Child Health Hospital College of Clinical Medicine for Obstetrics & Gynecology and Pediatrics, Fujian Medical University, Fuzhou, 350001 China; 2 Department of Pediatric Orthopedics, Fujian Children’s Hospital, Fuzhou, 350011 China; 3 Department of Orthopedics, Fuzhou Second Hospital Affiliated to Xiamen University, Fuzhou, 350007 China

**Keywords:** Distal tibia physeal, Fracture, Growth disturbance

## Abstract

**Background:**

To analyze the growth disturbance after distal tibia physeal fracture in children.

**Methods:**

Data about children with distal tibia physeal fractures between September 2015 to September 2018 were collected, including age, gender, affected side, Salter–Harris classification, initial maximal displacement, with or without fibula fracture, open or closed reduction, the method of fixation, time to surgery, blood loss, duration of operation, and complications. Patients were placed in the growth disturbance group when leg length discrepancy was equal to or greater than 1.5 cm, or when they had more than 5 degrees of varus or valgus deformity. Others were placed in the no-growth disturbance group.

**Results:**

A total of 143 patients (96 boys and 47 girls) were enrolled in this study. The length of the follow-up periods averaged 29.34 ± 7.46 months (26 to 61 months). Among the total of 143 patients, the incidence of growth disturbance was 15.39% (22/143). The no-growth disturbance group consisted of 121 patients (84 boys and 37 girls), with a mean age of 11.50 ± 3.20 years, and there were 68, 52, and 1 injuries to the left, right, and bilateral sides, respectively. The average maximal displacement was 5.51 ± 3.18 mm, and 27.27% (33/121) of patients also had a fibula fracture. The growth disturbance group contained 22 patients (12 boys and 10 girls) with a mean age of 9.32 ± 3.56 years, and there were 12, 10, and 0 injuries on the left, right, and bilateral sides, respectively. The average maximal displacement was 5.77 ± 4.89 mm, and 45.45% (10/22) of patients also had a fibula fracture. There was a significant difference in age (*p* = 0.004) and fibula fracture (*p* = 0.011) between the two groups. More patients had Salter–Harris types III and IV fractures in the growth disturbance group than in the no-growth disturbance group (*p* = 0.043).

**Conclusions:**

Children with Salter–Harris types III and IV fractures, younger children, and children with fibula fractures all have a higher incidence of growth disturbance after distal tibia physeal fractures.

**Level of evidence:**

Level III-Prognostic study.

## Introduction

Distal tibia physeal fractures are a relatively common physeal injury and are the second most common injury in children after the distal radius physis [1, 2]. Distal tibia physeal fractures account for approximately 11–20% of all physeal fractures [3, 4]. The stronger ligamentous attachments make the physis more vulnerable to injury and more likely to be accompanied by subsequent premature growth arrest, which causes angular deformity and differences in leg length 1–2 years after injury [1, 5, 6]. Growth disturbance is defined as differences in leg length equal to or greater than 1.5 cm or varus or valgus deformity more than 5 degrees [7]. According to different studies, the incidence of growth disturbance varies as between 2 and 40% [8–10].

Multiple factors, including the cause of injury, fracture type and location, initial displacement, Salter–Harris classification, number of attempted reductions, open or closed reduction, and residual displacement after the intervention, are thought to be associated with growth disturbance [6, 11–13]. However, the factors affecting growth disturbance are still unclear. Therefore, this study aims to assess the factors related to growth disturbance after distal tibia physeal fractures in children.

## Patients and methods

Data about children with distal tibia physeal fractures who were treated in Fuzhou Second Hospital Affiliated with Xiamen University between September 2015 to September 2018 were collected. All patient files were reviewed with the approval of the hospital. Patients were placed in the growth disturbance group when the difference in their leg lengths was equal to or greater than 1.5 cm or more than 5 degrees of varus or valgus deformity. Otherwise, they were placed in the no-growth disturbance group.

All patients were further divided into early and delayed operation groups. According to Rang and Salter [14, 15], patients were classified in the early operation group when the time from injury to operation was equal to or less than 7 days, and classified in the delayed operation group when it exceeded 7 days.

The following data were extracted from the database: age, gender, affected side, Salter–Harris classification, initial maximal displacement, the presence of fibula fracture, open or closed reduction, method of fixation, time to surgery, blood loss, duration of operation, and complications.

The inclusion criteria were as follows:

1. Age less than 16 years.
2. Diagnosed with distal tibia physeal fracture.
3. Residual displacement less than 2 mm after surgery, measured by scale with a 2 mm diameter steel ball or K-wire nest to the ankle joint.
4. Follow-up more than 12 months.

The exclusion criteria were as follows:

1. Peripheral vascular diseases.
2. Neuromuscular diseases.
3. Metabolic bone disorders.
4. Pathological fractures.
5. Residual displacement more than 2 mm after surgery.

SPSS 22.0 software was used for all statistical analyses. Descriptive statistics were conducted for continuous variables using mean ± standard error (SE). A comparison test between two independent or more than two groups was made using a two-sided Student’s *t*-test or one-way analysis of variance at a 5% significance level (2.5% on each side). Descriptive statistics of categorical variables were represented as frequency and percentage. A Chi-square test was used to compare the differences in rates among different groups at a 5% significance level (2.5% on each side).

## Results

A total of 143 patients (96 boys and 47 girls), with an average age of 11.19 ± 3.34 (range from 2.8 to 16) years, were enrolled in this study. The follow-up periods averaged 29.34 ± 7.46 months (26 to 61 months). The overall incidence of growth disturbance was 15.39% (22/143); among them, 21 cases had varus or valgus deformities greater than 5 degrees, and 1 case had a limb shortening exceeding 1.5 cm. However, no infection, nonunion, or osteonecrosis was observed in any patient.

The no-growth disturbance group contained 121 patients (84 boys and 37 girls), with a mean age of 11.50 ± 3.20 years, and 68, 51, and 1 cases on the left, right, and bilateral sides, respectively. The average maximal displacement was 5.51 ± 3.18 mm. The growth disturbance group contained 22 patients (12 boys and 10 girls) with a mean age of 9.32 ± 3.56 years, and 12, 10, and 0 cases on the left, right, and bilateral sides, respectively. The average maximal displacement was 5.77 ± 4.89 mm, and the average comprehensive displacement was 5.05 ± 2.38 mm. There was a significant difference in age between the two groups (*p* = 0.004), but no significant difference in the factors described above (Table 1).

**Table 1:** The data of patients between no-GD group and GD group

Group	no-GD group (*n* = 121)	GD group (*n* = 22)	*p* value
Gender (male/female)	84/37	12/10	0.172
Age	11.50 ± 3.20 (median 12)	9.32 ± 3.56 (median 11)	0.004
Initial maximal displacement (mm)	5.51 ± 3.18 (median 4)	5.77 ± 4.89 (median 4)	0.748
Fracture			0.011
Tibia	88	10	
Tibia and fibula	33	12	
Days between injury and surgery	4.73 ± 4.85 (median 3)	5.77 ± 6.47 (median 3)	0.38
Reduction			0.215
Open	44	5	
Close	77	17	
Operation duration (min)	40.87 ± 26.88	49.32 ± 66.21	0.561
Fixation of tibia			0.963
K-wire	44	8	
Screw	76	14	
Plate	1	0	

*GD* Growth disturbance

**Fig. 1:**
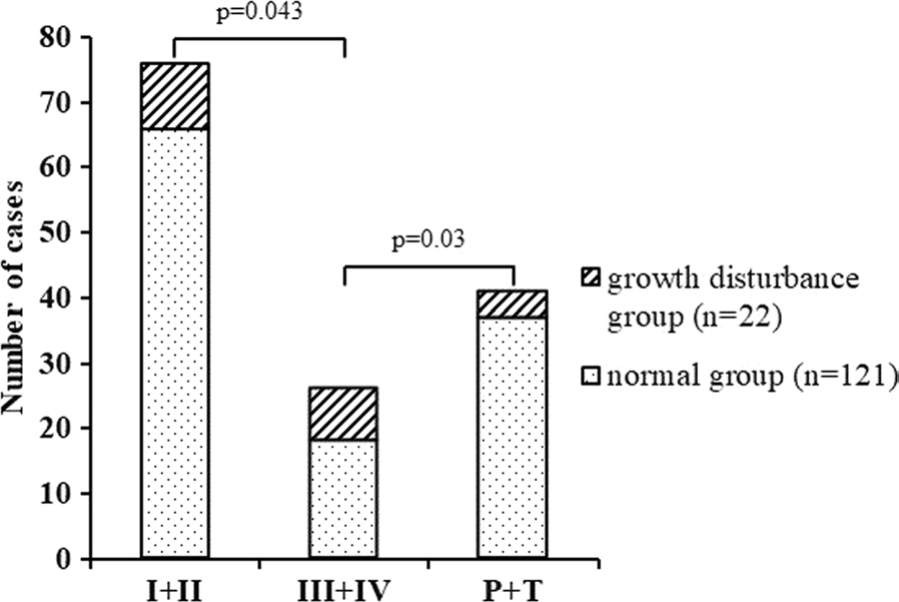
The risk of growth disturbance between Salter–Harris types. (P, Triplane fractures; T, Tillaux fractures)

According to Salter–Harris classification, patients with type I/II/III/IV fracture were 7/59/9/9 in the normal group and 3/7/4/4 in the growth disturbance group. As transitional fractures, there were 31 Triplane fractures and 6 Tillaux fractures in the no-growth disturbance group, and 4 Triplane fractures and 0 Tillaux fractures in the growth disturbance group (Table 2). More Salter–Harris types III and IV patients were in the growth disturbance group than in the no-growth disturbance group (*p* = 0.043) (Fig. 1). The SH grade analysis showed that growth disturbance occurred 3/10 (30%) in SH grade I, 7/66 (10.61%) in SH grade II, 4/13 (30.77%) in SH Grade III, and 4/13 (30.77%) in SH grade IV (Table 2).

**Table 2:** Patients whose fracture type was registered according to Salter–Harris classification and transitional fractures

Fracture type	no-GD group (*n* = 121)	GD group (*n* = 22)
Salter–Harris		
I	7	3
II	59	7
III	9	4
IV	9	4
Triplane	31	4
Tillaux	6	0
*p* value	0.69	

*GD* Growth disturbance

Among the no-growth disturbance group, 27.27% (33/121) of patients also had fibula fractures, while 45.45% (10/22) of patients in the growth disturbance group also had fibula fractures, which was significantly different (*p* = 0.011). Differences in open or closed reduction, type of fixation, duration of operation, and blood loss were not significant between the two groups. Forty-four cases received K-wires, 76 received screws, and 1 received plates in the no-GD group; 8 cases received K-wires, 14 received screws, and 0 received plates in the GD group. Differences between the two groups were not significant.

**Fig. 2:**
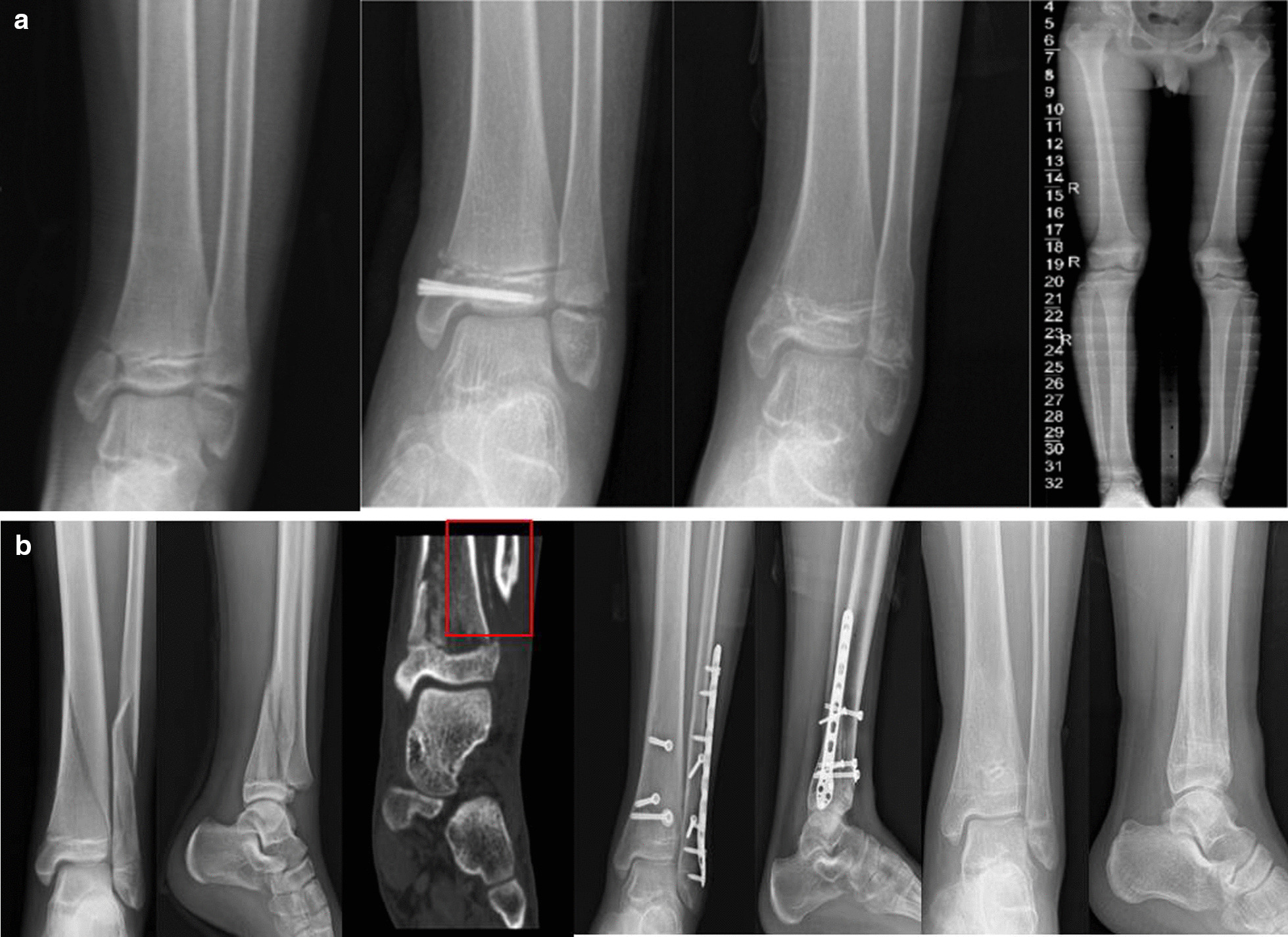
**a** An 11-year-old patient, Salter–Harris type II, distal tibia physeal fracture accompanied by fibula fracture, accepted open reduction 17 days after injury, osteotyluss growth can be seen in CT scan showed in the red frame. Growth disturbance has not been observed during 39 month follow-up. **b** An 11-year-old boy, Salter–Harris type III Distal tibia physeal fracture, accepted open reduction 4 days after injury. Growth disturbance has been observed at 13-month follow-up, the lateral angle of the distal tibia was 98° at 26-month follow-up

**Fig. 3:**
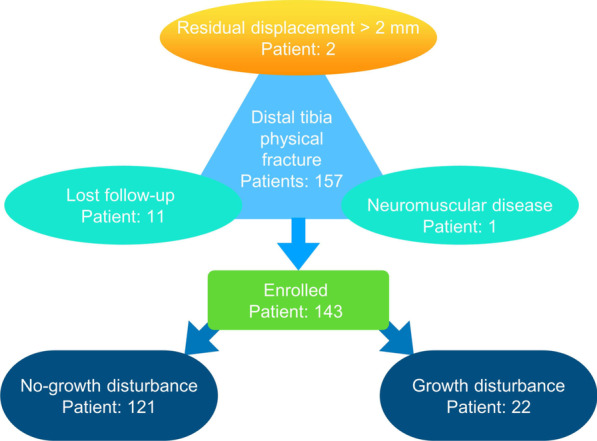
Flow diagram detailing the methods used to identify study subjects

There was no significant difference in growth disturbance between the early group (18/123) and the delayed operation group (3/20) (*p* > 0.05) (Figs. 2, 3).

## Discussion

Like some fractures, physeal injuries can be complicated by malunion, infections, neurovascular problems, or osteonecrosis. The incidence of growth disturbance after distal tibia physeal fractures was variable based on previously published findings, ranging from 2% [8] to almost 40% [9]. In this study, the overall incidence of growth disturbance was 15.39% (22/143), which was within the range of previous research [8, 9, 16].

Growth disturbance has been related to many different factors, including the cause of injury, fracture type and location, initial displacement, Salter–Harris classification, number of attempted reductions, open or closed reduction, quality of reduction, and the different composition characteristics of patients. However, few articles have reported the effects of age on growth disturbance after distal tibia physeal fractures. Our study found that age was an important factor affecting growth disorders. The average age of the growth disturbance group was significantly lower than that of the no-growth disturbance group. This could be due to the greater growth potential of younger children and a greater effect on growth after a fracture. Additionally, there were 4 Triplane fractures and 0 Tillaux fractures among the 22 patients with malformations, which could occur in older adolescents. This suggests that age was an important factor in growth disorders after distal tibia physeal fractures.

The growth disturbance in grade I was similar to that in grade III/IV, probably because there were so few cases in grade I. Salter–Harris types III and IV fractures displayed a higher growth disturbance, which could be due to injuries to the germinal layer of the physis [17]. Our study also found that there were more Salter–Harris types III and IV patients in the growth disturbance group than in the no-growth disturbance group (*p* = 0.043) (Fig. 1).

We also found that the growth disturbance group (12/22) (Table 3) was more likely to also have fibula fractures than the normal group (33/121) (*p* = 0.011). Patients with fibula fractures could have suffered more significant injuries. Significant injuries often manifest as larger initial displacements but can also be accompanied by other fractures.

**Table 3:** The data of patients in growth disturbance (GD) group

Case	Gender	Age (years)	Side	Types	Fixation of tibia	Fibula affected	Initial maximal displacement (mm)	Reduction	Operation duration (min)
1	Female	11	Right	III	Screw	NO	4	Close	10
2	Female	8	Right	I	K-wire	Yes	8	Close	75
3	Male	11	Left	II	K-wire	Yes	7	Close	20
4	Male	11	Left	Triplane	Screw	NO	4	Close	25
5	Male	8	Right	I	K-wire	Yes	4	Close	30
6	Male	13	Left	II	Screw	NO	4	Close	15
7	Female	11	Left	II	Screw	Yes	6	Open	70
8	Male	13	Right	Triplane	Screw	Yes	5	Open	80
9	Male	3.91	Left	II	K-wire	NO	2	Close	20
10	Female	11	Right	IV	Screw	Yes	3	Close	15
11	Male	12	Left	III	screw	NO	4	Open	40
12	Female	2.25	Left	II	Screw	Yes	4	Close	30
13	Female	5	Left	III	K-wire	NO	3	Close	45
14	Male	13	Right	Triplane	Screw	Yes	11	Close	25
15	Female	13	Left	Triplane	Screw	NO	5	Open	45
16	Male	12	Left	II	K-wire	Yes	10	Close	35
17	Female	2.8	Right	IV	Screw	NO	3	Close	10
18	Male	8	Right	I	Screw	NO	3	Close	25
19	Female	12.8	Right	IV	K-wire	NO	25	Open	330
20	Male	7	Right	III	K-wire	Yes	3	Close	30
21	Female	6	Left	II	Screw	Yes	3	Close	35
22	Male	9	Left	IV	Screw	Yes	6	Close	75

Patients were classified either into an early operation group or a delayed operation group based on the methods used by Rang and Salter [13, 14]. They suggested that delayed reduction makes it more difficult to achieve fracture reduction and places the viability of the physis at risk when forceful reduction is required. However, Egol KA reported that delayed reduction showed no evidence of physeal damage, physeal growth disturbance, or radiographic bar formation in a rat model study [18]. Our study included closed or open reduction in 20 delayed operation patients (mean 12.8 ± 6.68 days, range from 8 to 37 days). Differences in growth disturbance were not significant compared with early operation patients (Fig. 2).

This study had some limitations. It was a retrospective study, and the number of cases was limited. Growth disturbance from a physeal fracture is typically evident 2 to 6 months after the injury, but it may not become obvious for up to 2 or more years after the injury. Therefore, follow-ups should be extended to near skeletal maturity. However, Stenroos et al. [19] suggested that routine radiographic follow-up is unnecessary after physeal fractures of the distal tibia in children. We believe this study will significantly improve prognosis in children after distal tibia physeal fractures.

## Conclusion

Patients in the growth disturbance group were significantly younger and more likely to be Salter–Harris types III and IV than patients in the no-growth disturbance group. Patients who also had fibula fractures could have a higher incidence of growth disorders.

## Data Availability

The data used for analysis in our study are available from the corresponding author on reasonable request.
